# A pH-dependent molecular switch for virion uncoating

**DOI:** 10.1007/s13238-014-0094-4

**Published:** 2014-08-07

**Authors:** Satoshi Koike

**Affiliations:** Neurovirology Project, Tokyo Metropolitan Institute of Medical Science, 2-1-6 Kamikitazawa, Setagaya-ku, Tokyo, 156-8506 Japan

Enterovirus 71, belonging to human enterovirus species A in the genus *Enterovirus* of the family *Picornaviridae*, is one of the main causative agents of hand-foot-mouth disease. It can also cause severe neurological diseases such as acute encephalitis, polio-like paralysis, and neurogenic pulmonary edema, and it has become a serious public health concern especially in the Asia-Pacific region.

Native virions of enteroviruses are composed of a positive-stranded RNA genome and 60 copies of each capsid protein; namely VP1, VP2, VP3 and VP4. The natural lipid called “pocket factor” is harbored in the hydrophobic pocket of the VP1 capsid protein and plays an important role in stabilizing the native virion structure. Enterovirus infection is initiated by the binding of virions to their respective receptors followed by their internalization into the cells. Poliovirus receptor (PVR) and Coxsackie virus-adenovirus receptor (CAR) bind to a depression around the five-fold axes, called “canyon”, on poliovirus and coxsackie B viruses (CVBs), respectively. The internalized virion undergoes serial structural changes that lead to a release of viral genomic RNA into the cytoplasm. This step is called uncoating. After binding of the virion to the receptor, the “pocket factor” and the capsid protein VP4 are released from the virion. This uncoating intermediate is called “altered particle” (A-particle). In the A-particle, large holes are generated near the two-fold axes as result of a rearrangement of the capsid proteins. The genomic RNA is then released from the hole and the resulting particle is called “empty capsid”. The uncoating of poliovirus and CVB proceeds at neutral pH.

As for EV71, several cellular receptors for EV71 have been identified. Among these receptors, scavenger receptor class B member 2 (SCARB2) plays a pivotal role in EV71 infection. SCARB2 mediates binding of EV71 at the cell surface and internalization of the virus via a clathrin-dependent pathway. Finally, it initiates a conformational change that leads to uncoating. However, unlike the uncoating of poliovirus and CVBs, uncoating of EV71 requires the acidification of endosomal or lysosomal compartments. The mechanism underlying this uncoating has not been fully elucidated. In particular, the binding interfaces of SCARB2 and EV71 have not been determined, although SCARB2 is suggested to bind to the “canyon” of EV71 particles like other uncoating receptors, and importance of acidic pH conditions in establishment of infection was unclear.

The article by Dang et al. in this issue provides a structural basis for the uncoating process of EV71 and throws light on how SCARB2 and EV71 interact. Using peptides from the outer surface of the virion, they found that peptides that form the wall of the canyon bind to SCARB2. The GH loop of the capsid polypeptide VP1 strongly binds to SCARB2 and is proposed to act as a sensor-adaptor for SCARB2 binding. Although this is not direct structural evidence, the biochemical data strongly suggest that SCARB2 binds to the canyon of the virion like other uncoating receptors.

More importantly, they determined the three-dimensional structure of the ectodomain of SCARB2 at neutral and acidic pH conditions at 3.0 Å resolution. It is known that SCARB2 plays a physiologically important role in transporting newly synthesized β-glucocerebrosidase (β-GCase) to lysosomal compartments. β-GCase binds to SCARB2 in the endoplasmic reticulum at neutral pH and β-GCase dissociates from SCARB2 in lysosomes at acidic pH, suggesting that the conformation of SCARB2 under neutral and acidic pH conditions is different. The virus-binding domain as well as the β-GCase binding domain of SCARB2 mapped to the head (membrane-distal) region of SCARB2 and structural analysis revealed a marked difference in this domain in two conditions. Further results showed that a histidine residue at position 150 within the linker region of helices α4 and α5 acts as a pH sensor and protonation of this residue at acidic pH induces a rearrangement of this region. Under acidic pH conditions, helix α4 becomes shorter and α5 becomes longer in length as the new linker region folds away from α7. This structural rearrangement in the virus-binding domain of SCARB2 may trigger structural changes in the EV71 virion, probably by dislocating the sensor-adaptor region of the virion. The pocket factor may be dislodged by this rearrangement.

Members of the CD36 family of scavenger receptor proteins have hydrophobic lipid-transfer tunnels through the entire length of the ectodomain. The tunnel in Scavenger receptor class B member 1 (SR-BI) plays a role in cholesterol (ester) transportation between high-density lipoprotein (HDL) and the membrane. The lipid-transfer tunnel of SCARB2 is closed at the top by a cap formed by helices α5 and α7 under neutral pH conditions. However, displacement of these helices at acidic pH results in opening of the lid. According to the most probable docking model, the membrane-distal end of the lipid-transfer tunnel is in the vicinity of the hydrophobic pocket in VP1. The authors hypothesized that this lipid-transfer tunnel participates in expulsion of the “pocket factor” from the virion.

The results clearly show that SCARB2 acts as a pH-dependent molecular switch for the initiation of uncoating. In this respect, the study is a major contribution to understanding the early events of EV71 infection. However, several questions remain unsolved. For example, the precise nature of the interaction between the acidic form of SCARB2 and EV71 has not been clarified. Which amino acids are most important for the interaction? The authors discussed the importance of the lipid-transfer tunnel in SCARB2. However, what kind of structure in PVR and CAR, which have no obvious lipid-transfer tunnel, would complement this function? I expect that these questions will be solved by further studies including a structural analysis of the virus-receptor complex at high resolution.

